# A bibliometric analysis of Oropouche virus

**DOI:** 10.3389/fmicb.2024.1457773

**Published:** 2024-10-09

**Authors:** Jingsha Dong, Zichen Li, Shan Gao, Leiliang Zhang

**Affiliations:** ^1^Department of Clinical Laboratory Medicine, The First Affiliated Hospital of Shandong First Medical University and Shandong Provincial Qianfoshan Hospital, Jinan, Shandong, China; ^2^Department of Pathogen Biology, School of Clinical and Basic Medical Sciences, Shandong First Medical University and Shandong Academy of Medical Sciences, Jinan, Shandong, China

**Keywords:** OROV, bibliometrics, VOSviewer, CiteSpace, Oropouche fever

## Abstract

**Objectives:**

Oropouche virus (OROV) causes systemic infections including the nervous and blood systems, posing a significant and growing public health challenge. However, a comprehensive review of the bibliometric analysis of OROV is still lacking. Therefore, the objective of this study was to provide insight into the research dynamics and current hotspots of OROV.

**Methods:**

This study used bibliometric analysis to explore the current status of research related to OROV. 148 publications from 1961 to 2024 were retrieved from the Scopus database. Countries, authors, institutions, journals, references, and keywords were visualized using VOSviewer, CiteSpace, R studio, and Bibliometrix. Microsoft Excel was used for statistical analysis.

**Results:**

Brazil is the country with the highest number of publications, total cited frequency, and the most extensive international collaboration. The most popular journal in this field is the American Journal of Tropical Medicine and Hygiene. Instituto Evandro Chagas is the institution with the highest number of publications, and Eurico Arruda is involved in the highest number of publications. Keyword co-occurrence analysis showed that Oropouche bunyavirus, virology, bunyavirus, priority journal, and nucleotide sequence are the main research hotspots in this field.

**Conclusion:**

Our study provides a comprehensive overview of the research trends and key areas of focus in OROV. The field is currently experiencing rapid growth, as evidenced by the rising number of annual publications, which not only highlights increased research activity but also lays a solid foundation for further in-depth investigations. This trend offers valuable insights for developing effective strategies for outbreak prevention and control in public health. Presently, researchers are concentrating on the detailed study of Bunyavirus infections, employing both virological and genetic approaches to elucidate their complex pathogenic mechanisms.

## 1 Introduction

Oropouche virus (OROV) belongs to the family *Peribunyaviridae*, genus *Orthobunyavirus*, and is a single-stranded, negative-sense RNA virus enveloped in a spherical lipid membrane (Files et al., 2022; Kuhn et al., 2023). Initially isolated in 1955 from mosquitoes in Trinidad and Tobago, it was named after a patient in Vega de Oropouche, Trinidad (Anderson et al., 1961). OROV is the causative agent of Oropouche fever (OROF). Several outbreaks attributable to OROV have been documented in Latin America, including Brazil, Peru, and Ecuador (Anderson et al., 1961; Watts et al., 1997; Romero-Alvarez and Escobar, 2018; Gutierrez et al., 2020; Bonifay et al., 2023). Currently, OROV is the second most prevalent arbovirus after dengue virus in the Brazilian legal Amazon, indicating its wide geographical distribution and significant epidemiological impact (Tilston-Lunel et al., 2015a). By late 2022, the initial cases of Oropouche fever were identified in Roraima, a northern state in Brazil. The outbreak then quickly spread to the eastern region (Moutinho, 2024). As of August 27, 2024, Brazil has reported a total of 7,848 cases (Lorenz and Chiaravalloti-Neto, 2024; Moutinho, 2024). On May 27, 2024, Cuba's Ministry of Public Health confirmed the first case of Oropouche fever in Santiago province (Castilletti et al., 2024). Additionally, on July 25, 2024, Brazil's Ministry of Health reported two fatalities due to Oropouche fever in Bahia. On August 2, 2024, Pernambuco recorded its first case of vertical transmission of the Oropouche virus, which resulted in fetal death (Lenharo, 2024; Martins-Filho et al., 2024a). OROV remains a concern as an endemic virus in tropical and subtropical regions (Romero-Alvarez and Escobar, 2018).

OROV similarities with Orthobunyaviruses suggest that OROV particles have an envelope ~90 nm in diameter, featuring surface distribution of glycoproteins Gn (N-terminal glycoprotein) and Gc (C-terminal glycoprotein), and containing three ribonucleoprotein (RNP) complexes, with each RNA fragment binding to multiple copies of nucleocapsid (N) and large (L) proteins (viral RNA-dependent RNA polymerase) (Obijeski et al., 1976). Analysis of N gene sequences has classified OROV into four genotypes (I, II, III, and IV), with an average nucleotide difference of ~5% between genotypes (Vasconcelos et al., 2011; Travassos da Rosa et al., 2017).

OROV, transmitted to humans via the bite of infected mosquitoes, has emerged as a key vector facilitating transmission between populations inhabiting forested areas and nearby water bodies (Romero-Alvarez and Escobar, 2018; Feitoza et al., 2023). The spillover of the virus from animal hosts to humans has raised profound public health concerns (Ellwanger and Chies, 2021). In the transmission cycle of OROV, Parakou midges serve as vectors for various arboviruses, with their reliance on human and wild mammalian blood constituting a global public health challenge (Sakkas et al., 2018). While human-to-human transmission of OROF has not been documented, nonhuman primates like howler monkeys, capuchin monkeys, and velvet monkeys, as well as mammals like sloths, are considered potential hosts for OROV in the sylvatic cycle (Pinheiro et al., 1981b, 1982a; Tilston-Lunel et al., 2015b; Travassos da Rosa et al., 2017; Sakkas et al., 2018). Two primary transmission cycles sustain the virus in nature: the urban cycle and the sylvatic cycle (Anderson et al., 1961; Pinheiro et al., 1981a; Romero-Alvarez and Escobar, 2017; Sakkas et al., 2018). Anthropogenic activities and increased populations of *C. paraensis* in urban areas promote human-vector interactions, potentially increasing the likelihood of vector-borne diseases (Pinheiro et al., 1981b).

After being bitten by an OROV-infected midge or mosquito, an incubation period of 3 to 8 days typically occurs (Pinheiro et al., 1981b, 1982b; Mercer et al., 2003; Sakkas et al., 2018). Patients infected with OROV usually exhibit symptoms of acute febrile illness, including fever, headache, muscle pain, joint pain, chills, photophobia, rash, and dizziness, but severe cases are rare (Pinheiro et al., 1981a; Travassos da Rosa et al., 2017; Romero-Alvarez and Escobar, 2018; Vernal et al., 2019). Some patients may also experience hemorrhagic symptoms, including spontaneous hemorrhage, or neurological complications such as encephalitis and meningitis associated with OROV infection (Vasconcelos et al., 1989; Mourãão et al., 2009; Vasconcelos et al., 2009; Alvarez-Falconi and Ríos Ruiz, 2010; Sakkas et al., 2018; Chiang et al., 2021; Sciancalepore et al., 2022). Despite the absence of specific treatments or vaccines for OROV, managing symptoms and providing supportive care remain the best strategies (Sakkas et al., 2018). However, with increased interest in OROV vaccine development, several teams are progressing with the development of vaccine candidates using different approaches, such as inactivated viruses, live attenuated viruses, and recombinant protein vaccines (Files et al., 2022). Notably, about 60% of patients may experience a recurrence of these symptoms within 1 to 2 weeks of recovery (Sakkas et al., 2018).

Bibliometrics, as a computational and statistical methodology, focuses on in-depth quantitative and qualitative analyses of literature within specific research areas, using literature databases to assess the contributions and collaborations of authors, institutions, countries, and journals. This approach provides insight into the current state of a subject area, revealing evolutionary tracks and potential trends in scientific research. In the context of OROV research, we have leveraged bibliometric tools such as VOSviewer, CiteSpace, and Bibliometric R software to systematically highlight research advances in the field (Synnestvedt et al., 2005; van Eck and Waltman, 2010). By employing a robust research methodology and advanced visualization tools, this study aims to provide insights into the current state of the OROV field and comprehensively reveal its current research landscape and potential future directions.

## 2 Methods

### 2.1 Data collection

**Figure 1:**
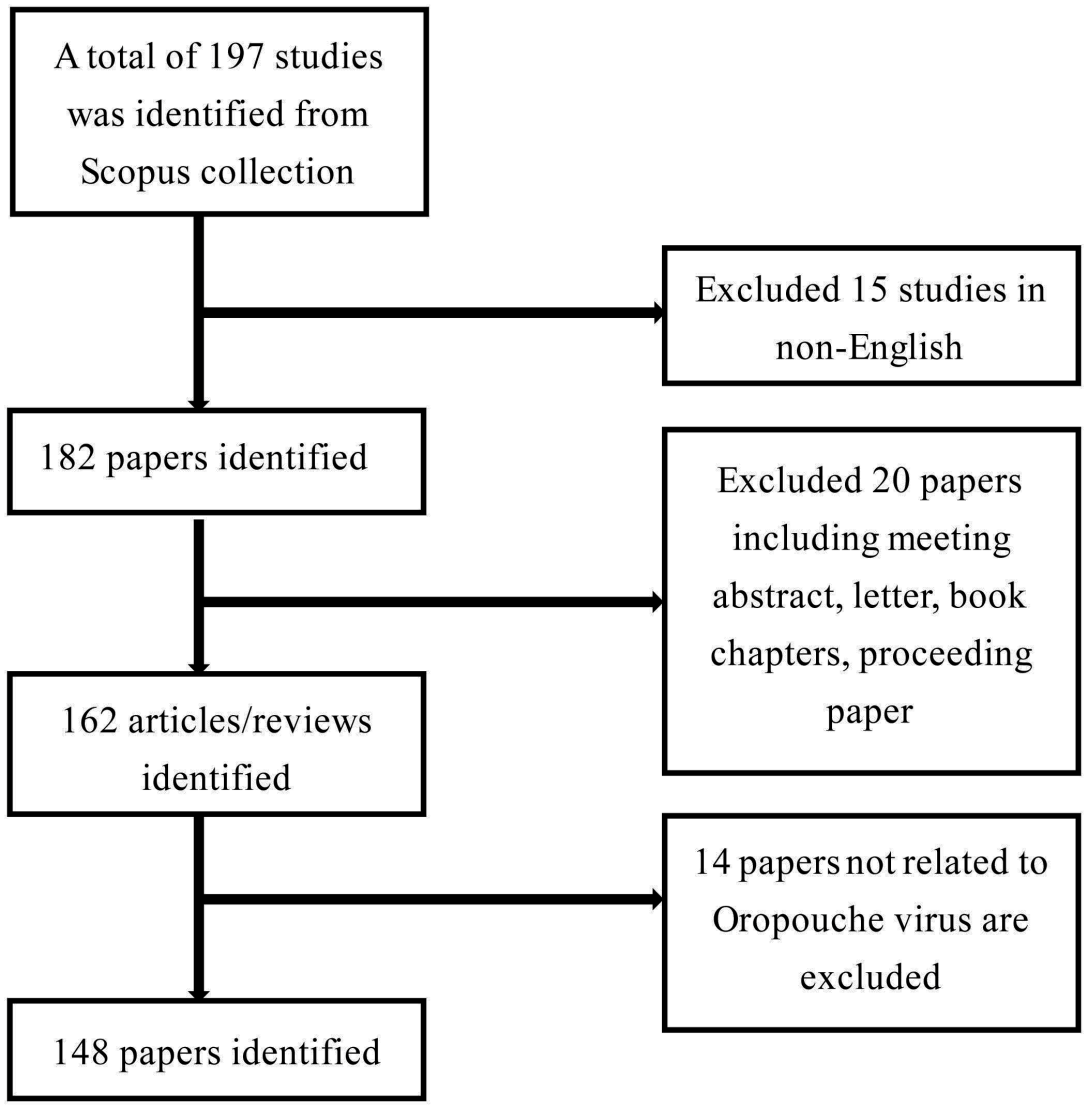
Flowchart of the screening process of publication on OROV.

In this study, the Scopus database served as the primary data source for bibliometric analysis. Scopus stands as a comprehensive, multidisciplinary abstract and citation database, globally acknowledged as a high-impact repository of scholarly resources, offering extensive coverage of scientific literature. The bibliometric analysis was conducted on April 26th, 2024. Utilizing Scopus, the study was searched using the Boolean expression (“oropouche virus” OR OROV), resulting in a total of 197 records. As illustrated in Figure 1, the initial results were refined as follows: (1) Filtered for documents in “English” (excluding 15 records). (2) Limited the document type to “articles” and “reviews” (excluding 20 records). (3) Excluded studies not related to the Oropouche virus (excluding 14 records). In the end, 148 studies were exported to a Comma-Separated Values (CSV) file in Microsoft Excel.

### 2.2 Data analysis and visualization

In this study, a variety of tools and software in scientometrics were employed for quantitative examination and analysis. VOSviewer (version 1.6.20), CiteSpace (version 6.3.R1), R Studio, Bibliometrix, and Microsoft Excel were utilized to analyze the study data from various angles. Microsoft Excel was used to measure and quantitatively analyze articles, countries, authors, and journals.

VOSviewer, a free tool, was utilized for managing and visualizing knowledge structures, creating bibliometric networks, and generating maps based on data such as country partnerships, author collaborations, and journal relationships. This aided in identifying research hotspots within the field by extracting keywords from bibliometric co-occurrence analysis.

CiteSpace, developed by Professor Chen at Drexel University, is a Java-based information visualization program designed for bibliometric analysis. This program enables researchers to visually identify leading-edge trends by presenting data in the form of a knowledge graph. Visual mappings of authors for each period were created using CiteSpace, where node types and sizes represent the weight of each parameter, and connecting lines indicate the strength of relationships between parameters. Timezone view analysis was also conducted to pinpoint research hotspots and trends.

Bibliometric analysis was conducted using R Studio version 2023.12.1 and the Bibliometrix tool version. This online analyzing platform facilitated the display of country cooperation on maps, identification of affiliate contributions, determination of the most cited literature and keywords, and generation of keyword clouds and treemaps.

## 3 Results

### 3.1 The trend of publication outputs

**Figure 2:**
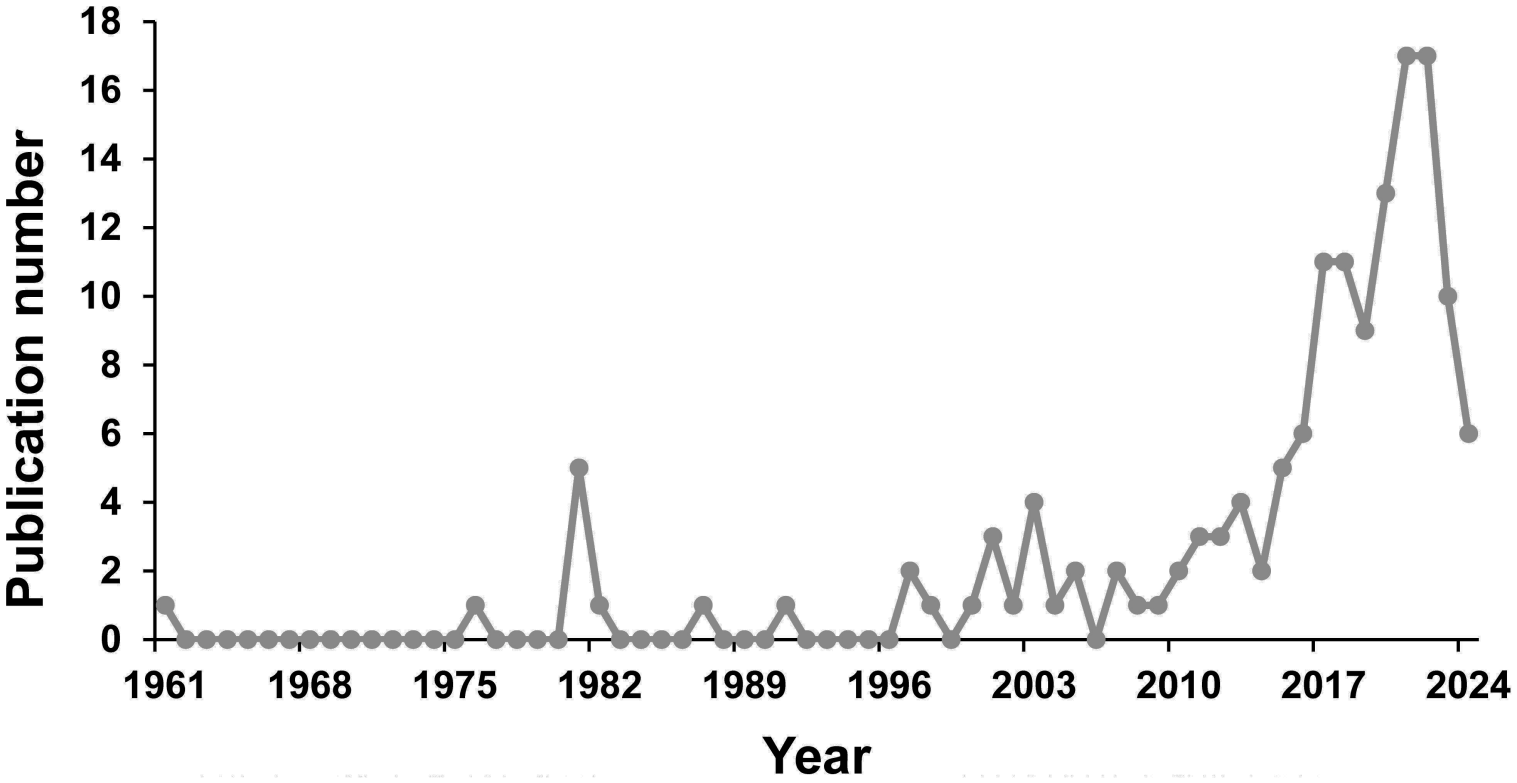
Annual number of publications on OROV from 1961 to 2024.

Based on the inclusion criteria of publication type and language, a total of 148 papers were included in this study for further bibliometric analysis. The temporal trend and the number of papers related to OROV are illustrated in Figure 2. From 1961 to 1996, there were fewer articles published, with a maximum of only one article per year. The relatively high number of publications in 1981 (*n* = 5) was attributed to the publication of a review covering the clinical, epidemiological, and ecological aspects of OROV, as well as the report of a series of epidemiological investigations into the 1975 OROV outbreak in Santarem. From 1997 to 2009, the number of annual publications remained relatively stable, ranging from 0 to 4 publications per year. Subsequently, from 2010 to 2022, there was a continuous increasing trend in the number of annual publications, except for 2014 and 2019, reaching a peak of 17 publications in both 2021 and 2022. However, from 2023 to 2024, there is a decreasing trend in the number of published articles.

### 3.2 Distribution of countries

**Figure 3:**
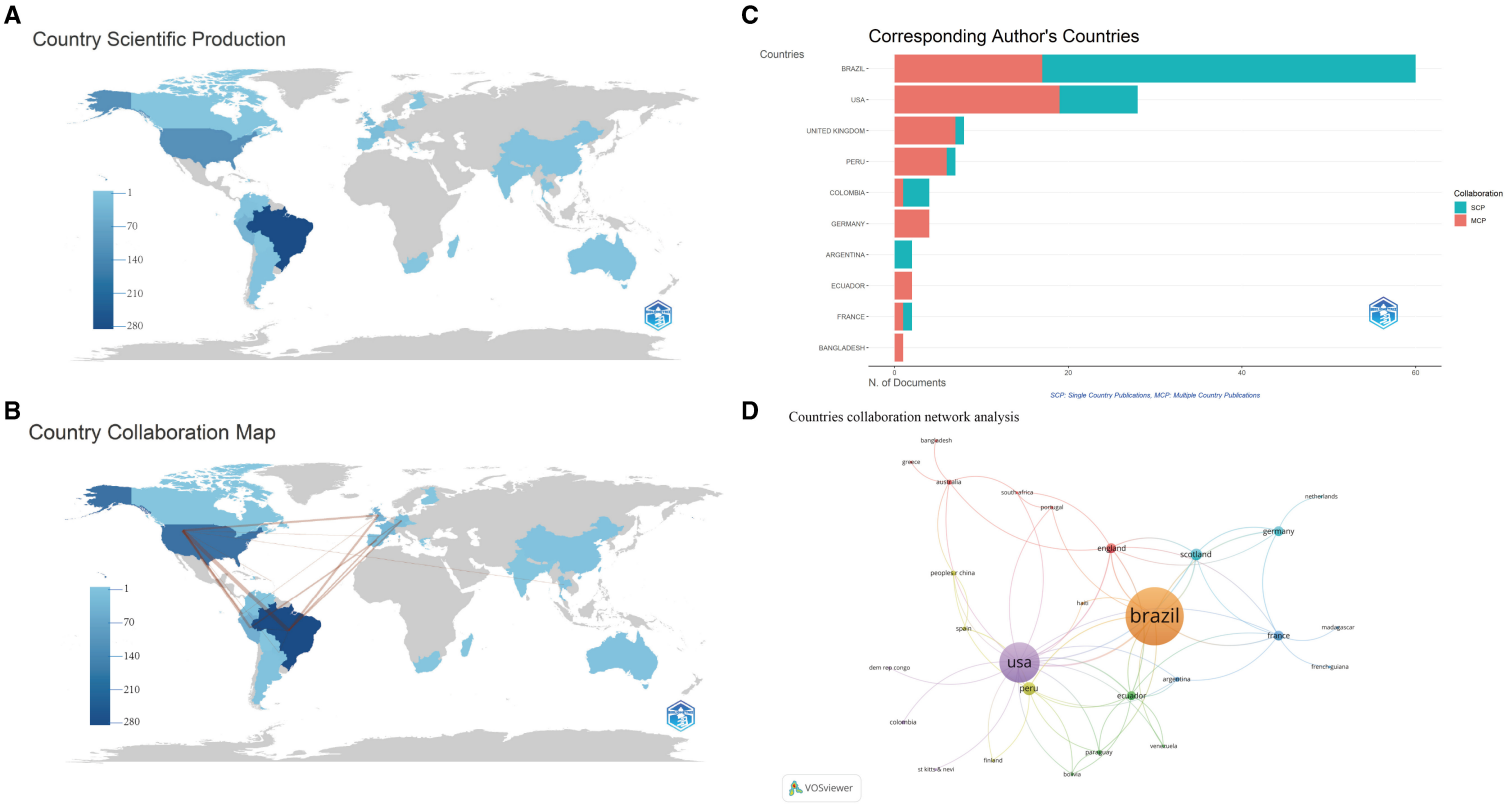
Visualization and analysis of the international collaboration networks in OROV research. The geographical distribution **(A)** and collaboration visualization **(B)** of countries and areas on the research of OROV. The choropleth map shows the geographical distribution of the cooperating countries. The change in color on the map (from light blue to dark blue) reflects the number of publications in each country. The number of any two countries connected by a red line is a direct reflection of the intensity of the cooperation between them. **(C)** The analysis of country co-authorships in the study, SCP, indicates that the authors of this paper are all from the same country, and MCP indicates that the authors of this paper are from different countries, where there is international collaboration. SCP, single country publications; MCP, multiple Country Publications. **(D)** Countries collaboration network analysis. Each node represents a country. The size of the node represents the number of publications, and the thickness of the connection between the nodes represents the intensity of cooperation.

A total of 28 countries have conducted relevant studies. Table 1 illustrates the top 10 countries that are globally productive. In terms of publication numbers, Brazil leads significantly with 90, followed by the United States with 58. As depicted in Figure 3A, Brazil's literature output in the field of OROV research reached 624 articles, this number significantly surpasses that of the United States (*n* = 255) and Peru (*n* = 94). Upon closer examination, the country cooperation network map reveals strong connections between countries like Brazil, the United States, Peru, the United Kingdom, Germany, and Spain, demonstrating extensive collaborative research efforts in this area (Figure 3B). The analysis indicates that Brazil leads in Single-Country Publications (SCPs), whereas the United States excels in Multi-Country Publications (MCPs), illustrating Brazil's dominance in OROV research and the United States' involvement in collaborative research with other nations (Figure 3C).

**Table 1:** Top 10 productive countries in the field of Oropouche virus.

**Rank**	**Country**	**Documents**	**Citations**	**Average citation per paper**
1	Brazil	90	2,103	23
2	United States	58	1,768	30
3	Peru	18	554	31
4	United Kingdom	13	492	38
5	France	10	500	50
6	Germany	7	249	36
7	Ecuador	6	272	45
8	Spain	6	88	15
9	Colombia	5	27	5
10	Paraguay	4	259	65

In this study, a co-authorship network analysis was conducted using VOSviewer software to map collaboration between countries. The minimum document count parameter for a country was set to 1, encompassing all 28 countries. Figure 3D displays the intensity of collaboration in OROV research among countries, with Brazil exhibiting an extensive network of international collaborations, particularly with the United States. Furthermore, Figure 3D highlights the significant presence of Brazil and the United States in the current research landscape. These visualizations offer valuable insights into collaborative networks and the substantial contributions of countries in OROV research.

### 3.3 Distribution of authors and co-cited authors

**Figure 4:**
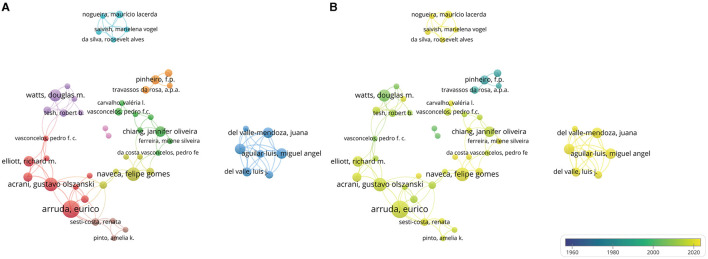
Visualization of author analysis. **(A)** Author collaboration network analysis. Minimum number of documents per author ≥3. The size of the node represents the number of author publications, and the thickness of the connection between the nodes represents the intensity of cooperation. **(B)** Cooperation time chart of author collaboration. Early occurrences of author collaborations are indicated by nodes labeled in purple or blue, and current author collaborations are indicated by nodes labeled in yellow.

**Figure 5:**
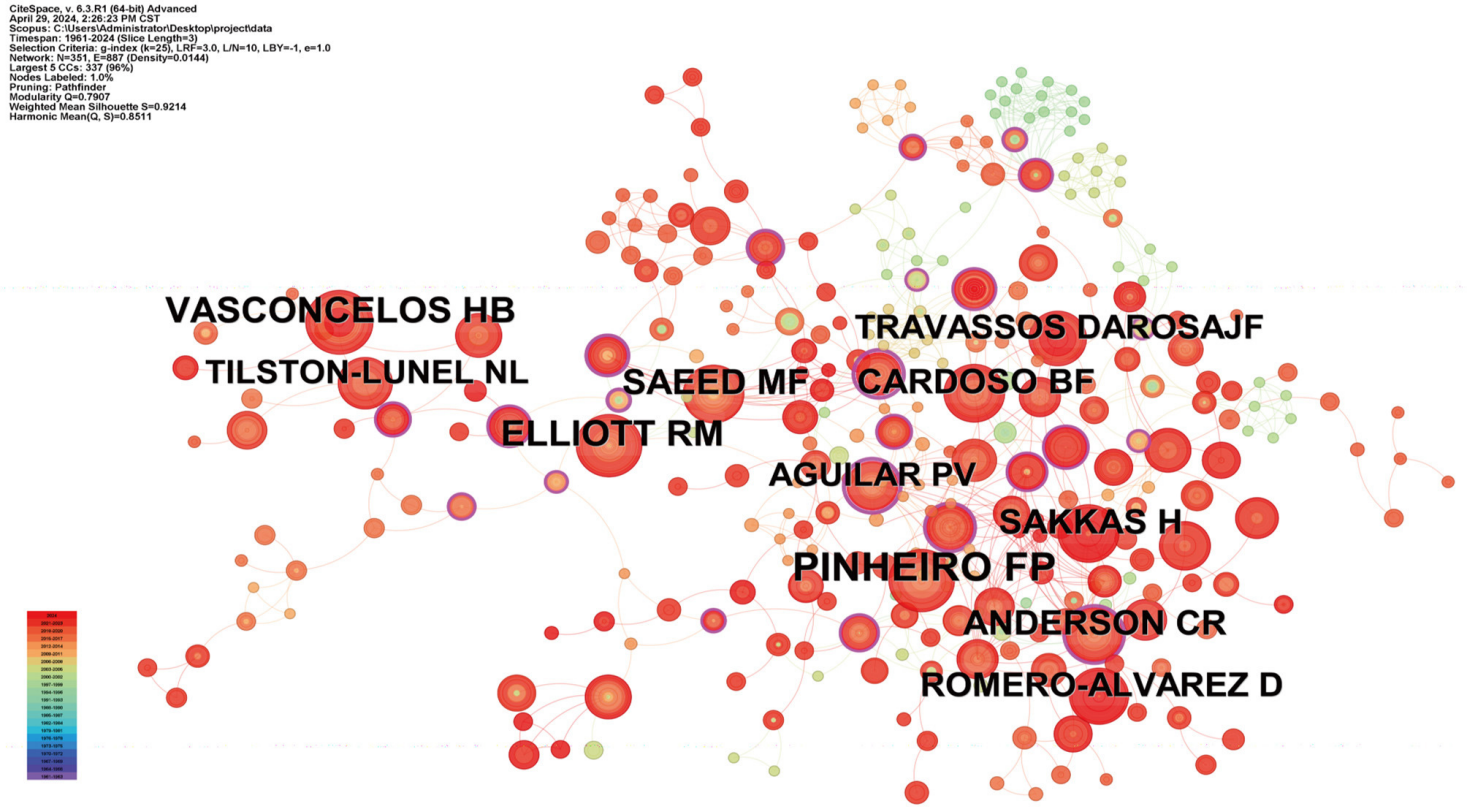
Co-citation of cited authors. The size of the circle indicates the number of cited papers, and the color of the link indicates the level of collaboration between the cited papers.

Over 900 authors have contributed to OROV research. Table 2 provides details on the 10 most productive authors in the field. Notably, Eurico Arruda leads the list with 11 articles, followed by Gustavo Olszanski Acrani and Felipe Gomes Naveca, each with 8 articles, showcasing their significant impact. The networks formed by these authors and their collaborators not only highlight prominent scholars in OROV research but also offer valuable insights into collaborative relationships, crucial for future advancements. Figure 4A illustrates the collaborative network among authors, with each author contributing at least 3 documents, resulting in 9 clusters and 51 nodes. Figure 4B zooms in on the collaborations among current authors, with Eurico Arruda exhibiting the most collaboration, denoted by the green node. Together with their peers, these prolific authors have enriched the breadth and depth of OROV research, emphasizing the collaborative nature of scientific exploration in the field. Furthermore, in Figure 5, we conducted citation analysis using CiteSpace (version 6.3.R1) software, focusing on authors cited within 2-year time slices. The analysis revealed Pinheiro FP as the most cited author (*n* = 68), followed by Vasconcelos HB (*n* = 42), Elliott RM (*n* = 41), and Saeed MF (*n* = 38).

**Table 2:** Top 10 productive authors in the field of Oropouche virus.

**Rank**	**Author**	**Documents**	**Citations**	**Average citation per paper**
1	Arruda, Eurico	11	206	19
2	Acrani, Gustavo Olszanski	8	268	34
3	Naveca, Felipe Gomes	8	250	31
4	Watts, Douglas M.	7	211	30
5	Aguilar-Luis, Miguel Angel	6	79	13
6	Del Valle-Mendoza, Juana	6	79	13
7	Silva-Caso, Wilmer	6	79	13
8	Elliott, Richard M.	6	193	32
9	Chiang, Jannifer Oliveira	6	178	30
10	Pinheiro, F.P.	5	194	39

### 3.4 Distribution of the affiliations

**Figure 6:**
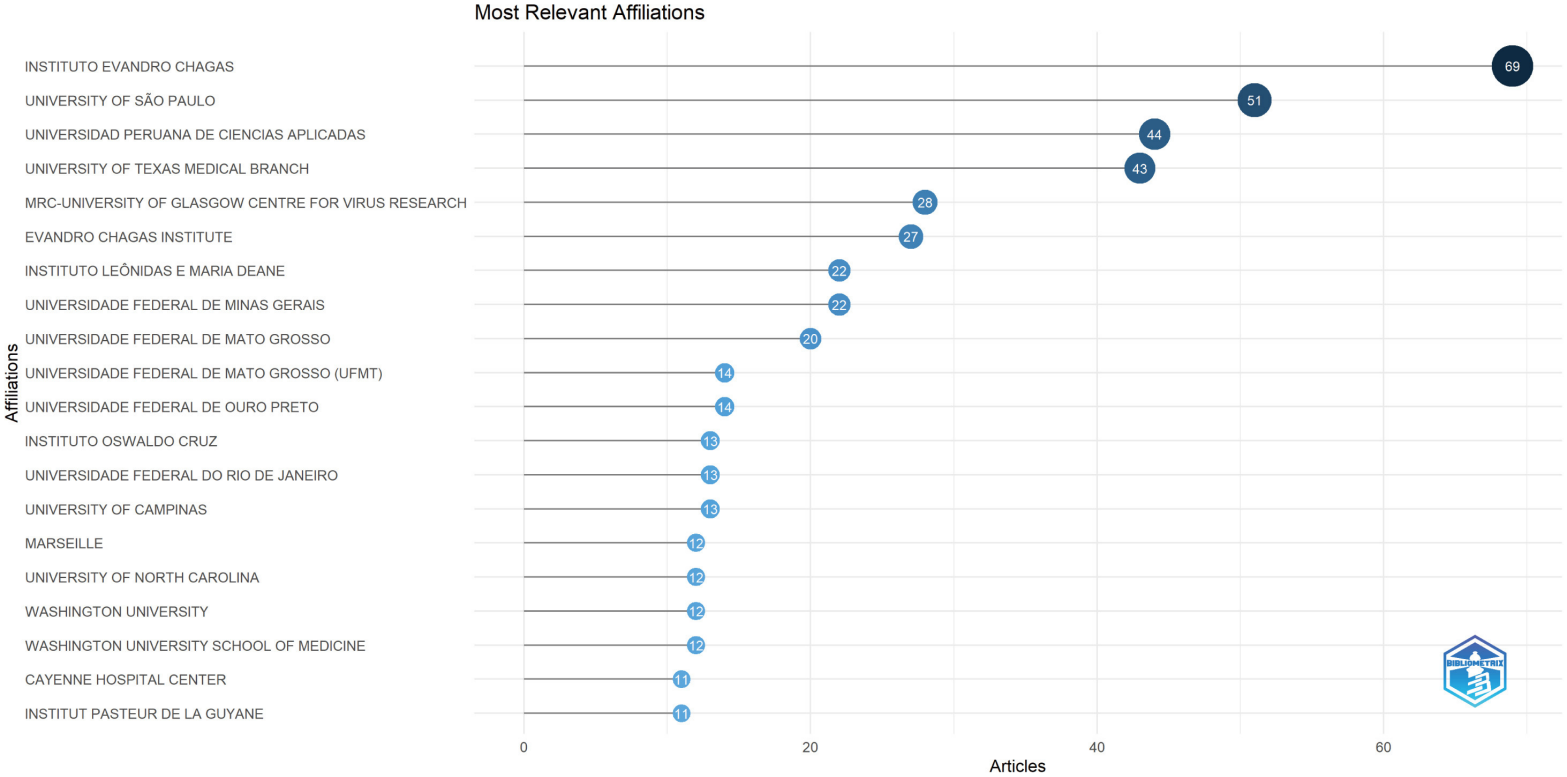
Top 20 most relevant affiliations on the research of OROV.

In Figure 6, we present the 20 highly productive institutions that have garnered significant recognition in OROV research. Instituto Evandro Chagas leads the list with the highest number of articles received and published (*n* = 69), closely followed by the University of São Paulo (*n* = 51) and Universidad Peruana de Ciencias Aplicadas (*n* = 44). Remarkably, half of the top 20 institutions are based in Brazil, followed by the United States and France. The outstanding performance of these institutions, as evidenced by various metrics, underscores their substantial influence and significance in the field.

### 3.5 Journals and co-cited journals

**Figure 7:**
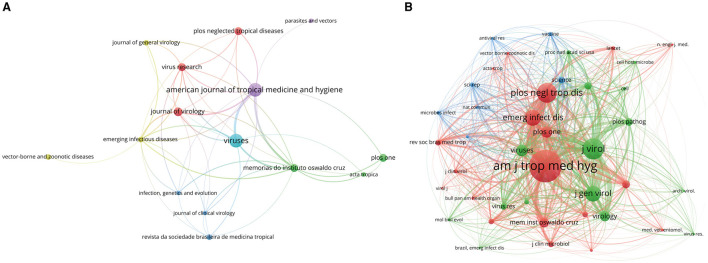
Analysis of journals involved in OROV. **(A)** The visualization of journals. Minimum number of documents per journal ≥3. As the largest node in the graph, the Journal of American Journal of Tropical Medicine and Hygiene has the most publications. **(B)** The visualization of co-cited journals. Minimum number of citations per journal ≥16. The size of the node is proportional to the frequency of co-citation, American Journal of Tropical Medicine and Hygiene has the most co-citations.

In the realm of OROV research, Table 3 outlines the top 10 journals making significant contributions, along with the top 10 co-cited journals. The American Journal of Tropical Medicine and Hygiene and Viruses leads the pack with 12 publications each, underscoring their pivotal role in advancing the field. Following closely is the Journal of Virology, with seven articles. Figure 7A presents a visual analytic graph depicting the journals, where node size corresponds to the number of published articles, and line thickness indicates the strength of collaborations between journals.

**Table 3:** Top 10 productive journals and co-cited journals in the field of Oropouche virus.

**Rank**	**Journals**	**Documents**	**Citations**	**Average citation per paper**	**Co-cited journals**	**Co-citations**
1	American Journal of Tropical Medicine and Hygiene	12	479	40	American Journal of Tropical Medicine and Hygiene	435
2	Viruses	12	206	17	Journal of Virology	218
3	Journal of Virology	7	226	32	Journal of General Virology	164
4	Memorias do Instituto Oswaldo Cruz	6	151	25	PLoS Neglected Tropical Diseases	128
5	Virus Research	6	126	21	Viruses	126
6	PLoS Neglected Tropical Diseases	6	231	39	PLoS One	123
7	PLoS ONE	6	118	20	Virology	109
8	Emerging Infectious Diseases	4	168	42	Emerging Infectious Diseases	86
9	Journal of General Virology	4	112	28	Nature	72
10	Vector-Borne and Zoonotic Diseases	4	133	33	Science	65

Regarding co-cited journals (Table 3), the American Journal of Tropical Medicine and Hygiene tops the list with a substantial co-citation frequency of 435, followed by the Journal of Virology with 218 co-citations, and the Journal of General Virology with 164. Figure 7B illustrates a visual network of 41 journals with more than 16 co-citation frequencies, with node size directly reflecting the frequency of co-cited articles. These findings serve as valuable guidance for researchers in selecting appropriate journals for publication, while also shedding light on the prominence and impact of journals in OROV research.

### 3.6 Reference analysis with strongest citation bursts

**Figure 8:**
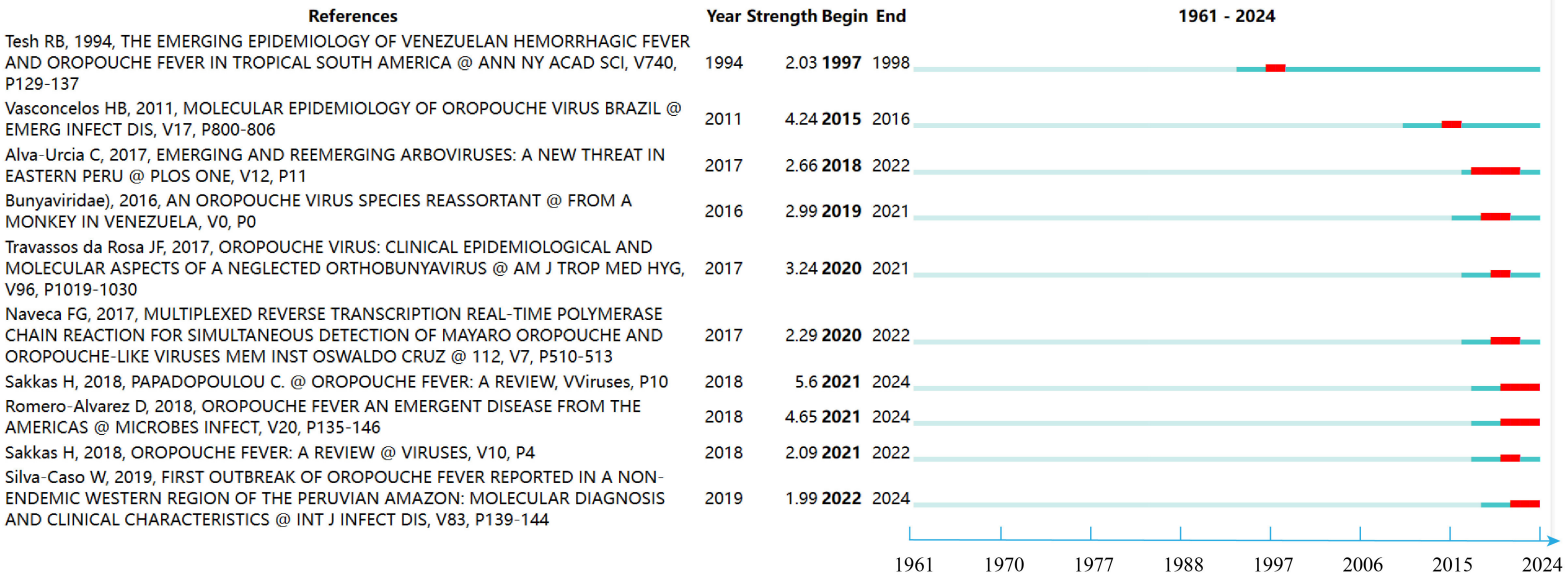
The top 10 references with the strongest citation bursts on OROV. The line represents the period and the red line represents the burst of strong citations.

Figure 8 illustrates significant papers alongside their respective active time spans, with distinct lines denoting different periods, and prominent red lines marking intense citation bursts. Among these, the review article “*Oropouche Fever: A Review*” by Sakkas H. et al., published in 2018, garnered substantial attention and citations between 2021 and 2024. This literature underscores OROF as the second most common arboviral febrile illness in Brazil, following dengue fever. Transmission primarily occurs through cycles involving both urban and forest environments, with the mosquito serving as a crucial vector in urban transmission. Despite extensive research, specific antiviral therapy for OROV remains elusive. Moreover, there's a looming risk of its further spread across the Americas, particularly under favorable climatic conditions, with potential expansion to other continents. The second notable burst of citations pertains to “*Oropouche fever, an emergent disease from the Americas*,” published by Romero-Alvarez and Escobar (2018), which also gained prominence during the 2021–2024 period. This literature highlights the challenge of distinguishing OROF symptoms from those of other arboviral diseases prevalent in the Americas. Furthermore, it emphasizes the potential exacerbation of OROV emergence due to habitat loss in South America shortly. Lastly, the third noteworthy literature is “*Molecular Epidemiology of OROV, Brazil*,” which gained prominence from 2015 to 2016.

### 3.7 Analysis of keywords

**Figure 9:**
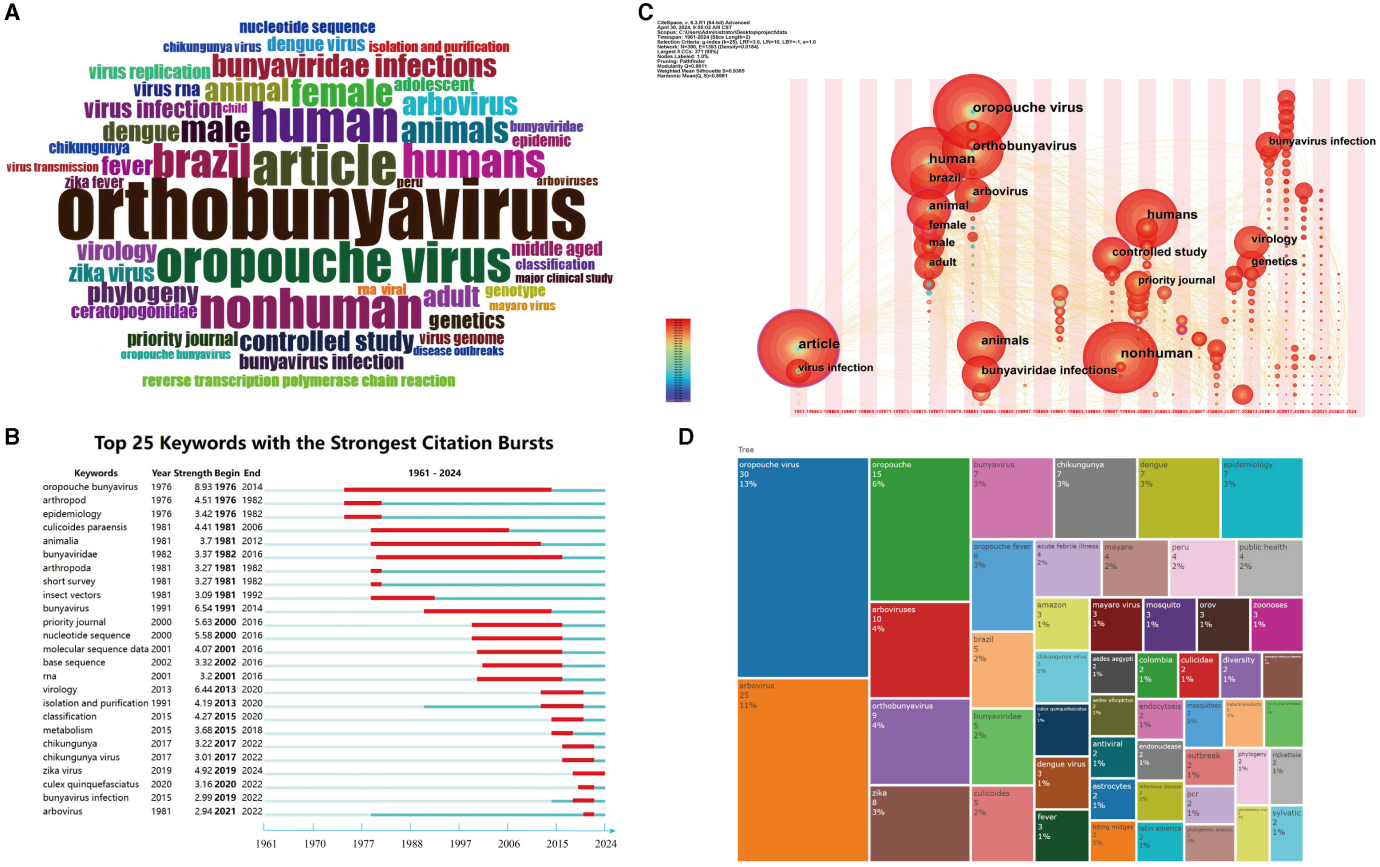
Visualization of keyword analysis. **(A)** Word cloud map of keywords in OROV. Word size represents the frequency of occurrence of the keyword. **(B)** The top 25 keywords with the strongest citation bursts on OROV. The line represents the period and the red line represents the burst of strong citations. **(C)** The time zone visualization map of co-occurring keywords in OROV. The timeline keywords indicate the initial appearance of each keyword, with node size reflecting its frequency. The connecting lines show common connections, and time is grouped in two-year intervals. **(D)** The clustering tree map of OROV. The size of the squares represents the frequency of occurrence of the keyword.

The role of keywords is pivotal in accurately summarizing the core content and theme of research. As depicted in Figure 9A, the keyword cloud map visually represents the frequency of keywords, intuitively unveiling the study's focus. The labels within the cloud map indicate the extent of researchers' attention to specific topics, with the frequency of keyword usage reflected by the number of labels; higher frequencies correspond to more labels. This figure highlights the central themes of orthobunyavirus, article, oropouche virus, human, and nonhuman.

Figure 9B illustrates the evolution of keywords, showcasing the top 25 keywords with the highest number of citation bursts. Each line represents a distinct period, with red lines denoting strong citation bursts. Notably, keyword citation bursts span from as early as 1976 to as late as 2024. The first cited keyword was “oropouche bunyavirus”. The second most cited keyword, “arthropod” ceased in 1982.

Figure 9C depicts the research trend of OROV through a time zone view. The movement of nodes from left to right signifies changes in researchers' focus on specific topics over time, with the size of each node reflecting researchers' levels of interest in the respective topics. From 1976 to 1977, themes like human, Brazilian, animal, and female dominated. In 1981-1982, research attention shifted to OROV, orthobunyavirus, arbovirus, animals, and bunyaviridae infections, among other topics. During 1997–2001, themes such as nonhuman, humans, controlled study, and priority journals garnered significant attention. The period of 2013–2015 saw increased emphasis on topics like virology, genetics, and bunyavirus infection.

Lastly, Figure 9D focuses on the frequency of keywords used by authors, with “oropouche virus” (*n* = 30, 13%), “arbovirus” (*n* = 25, 11%), “oropouche” (*n* = 15, 6%), “arboviruses” (*n* = 10, 4%), and “orthobunyavirus” (*n* = 9, 4%) among the most frequently used words.

## 4 Discussion

In academic research, bibliometric analysis has become an indispensable tool for providing a broad overview of the field and accurately capturing the trends and hotspots within it. Especially in the study of emerging and re-emerging viral diseases, bibliometric analyses of relevant publications are particularly important as they can provide insights to the global scientific and health communities on outbreak prevention and control strategies. This study is based on the widely recognized Scopus database with superior data quality and accuracy as a source of literature data, which covers a wide range of publications in the biomedical field and provides enriched features for bibliometric analysis (Burnham, 2006). To analyze in deep the research dynamics related to OROV, quantitative analysis software such as VOSviewer, CiteSpace, R studio, and Bibliometrix were used in this study. With these tools, 148 documents published in 72 academic journals from 1961 to 2024 were analyzed with 949 authors from 589 institutions in 28 countries/regions. The results of the study show that the amount of research literature on OROV shows a continuous increase over time, especially peaking in 2021 and 2022. The dynamics of this trend highlight the dynamic growth of this research area and may reflect continued advances in technology and research methodology. In addition, collaborative networks in research demonstrate the interdisciplinary quality of the field, while specific journals, prolific authors, and influential research institutions contribute significantly to the development of the academic field.

After an in-depth statistical analysis of the number of papers published by countries/regions in the field of OROV research, we found that although the total amount of relevant literature is not huge, certain key countries/regions have a significant influence in the field and their collaborative relationships are visible. Of these, Brazil and the United States serve as the main research forces, with Brazil taking the lead in terms of the number of publications. It is worth mentioning that more than half of the top 20 affiliates most relevant to research on the virus are located in Brazil, followed by the United States and France. It is noteworthy that the intensity of OROV infections is particularly prominent in Brazil. This phenomenon may reflect the fact that international academic development in the field of virus research is somewhat limited by geographic location. To promote the in-depth development of OROV research, we call on academic institutions to strengthen cross-regional and cross-country cooperation and exchanges to jointly address this global public health challenge. Brazil's significant contribution to this field undoubtedly deserves our further attention and study. At the same time, we have observed close cooperation in research on the virus between Brazil and countries such as the United States, Peru, the United Kingdom, Germany, and Spain.

At the level of research institutions, Instituto Evandro Chagas is particularly strong, topping the list, followed by the University of São Paulo and Universidad Peruana de Ciencias Aplicadas. This data not only highlights Brazil's significant strengths in the field of OROV research but also maps the collaboration between Brazil and other countries in the field of scientific research, especially with the United States. Despite the existence of cooperative relationships, the frequency, scope, and depth of inter-agency cooperation could be improved. For example, cooperation between Brazilian and United States institutions is still insufficient, which to some extent constrains development in this area. Because of the significant advantages of Instituto Evandro Chagas in terms of the number of publications and its scientific strength, we strongly recommend that research organizations in various countries strengthen their cooperation and communication with each other to promote the further development of the research field of OROV. By taking advantage of each other's strengths, we hope to achieve greater breakthroughs in this field, and at the same time break down academic barriers and build a more solid foundation for in-depth research on OROV.

From the point of view of authors' contribution, Eurico Arruda, Gustavo Olszanski Acrani, and Felipe Gomes Naveca are among the top three authors in terms of the number of articles published and the significant impact of their research in the field, as well as the total citation frequency, which is a good proof of their importance in the study of OROV. The latest findings from Eurico Arruda's team show that human nerve cells can support the production of infectious viral particles through OROV infection. In addition, OROV infection triggered the release of the pro-inflammatory cytokine TNF-α (tumor necrosis factor-α) and led to a decrease in cell viability within 48 hours of infection, suggesting that OROV is capable of inducing an inflammatory response and tissue damage. The research data not only reveal the neuropathogenesis of OROV, but also help to improve the understanding of the possible acute and chronic consequences of OROV infection in the human brain (Almeida et al., 2021). On the other hand, Pinheiro FP has the highest number of co-citations. His latest review provides a comprehensive review of the epidemiology, pathogenesis, and molecular biological characterization of OROV. It covers key information such as the first isolation of the virus, outbreaks over the past six decades, clinical expression of infection, diagnostic techniques, genomic and genetic characterization, evolutionary history, and viral transmission pathways, providing valuable insights into the current state of OROV research (Anderson et al., 1961).

The research on OROV has been published in two journals, “American Journal of Tropical Medicine and Hygiene” (IF = 3.3) and “Viruses” (IF = 4.7), which are undoubtedly the most productive academic platforms in this field. From the perspective of co-cited journals, “American Journal of Tropical Medicine and Hygiene” is still the most influential journal in the field. These journals provide strong support for OROV research with their high-quality content and international outlook. It is worthwhile for researchers in related fields to use them as the first-choice journals for submission and reference.

“*Oropouche Fever: A Review*” demonstrated the highest intensity of citation bursts, topping the list with an intensity of 5.6. The review was written in 2018 by Sakkas H et al. The authors are from the Department of Microbiology, Faculty of Health Sciences, School of Medicine, University of Ioannina, and the Department of Physiology, Anatomy and Microbiology, La Trobe University. This literature provides a comprehensive review of current research advances in OROV fever and the knowledge gaps that exist (Sakkas et al., 2018). This was closely followed by “*Oropouche fever, an emergent disease from the Americas*” by Romero-Alvarez and Escobar (2018), which came in second place with a slightly lower citation intensity. This article highlights the difficulties in distinguishing OROF from other febrile diseases caused by arboviruses in the Americas. Warning that habitat loss could elevate the risk of OROV outbreaks in South America in the near future (Romero-Alvarez and Escobar, 2018). Third in terms of citation intensity was the study “*Molecular Epidemiology of OROV, Brazil*” by Vasconcelos HB et al. from the Instituto Evandro Chagas. This study provides insight into the molecular epidemiology of OROV, revealing that each RNA segment has a distinct evolutionary history. The importance of comprehensively considering the genetic information of all genetic segments when classifying genotypes is emphasized. In particular, genotype I (based on N-gene data) is responsible for the emergence and viral transmission of all other genotypes (Vasconcelos et al., 2011).

In terms of keywords, orthobunyavirus, oropouche virus, human, nonhuman, and Brazil are by far the most popular topics favoring further research. The main research hotspots in this field include Oropouche bunyavirus, virology, bunyavirus, priority journal, and nucleotide sequence. It is hoped that the work in this paper will provide new ideas to advance the scientific research and clinical application of OROV.

Recently the first confirmed case of OROF in Rio de Janeiro on February 29, 2024, marked a further spread of the virus to a wider region beyond the confines of the traditional outbreak areas of Amazonas, Acre, and Rondônia. This development poses a serious test for the public health system and requires us to be vigilant in order to ensure the accurate identification and effective control of diseases (Martins-Filho et al., 2024b). Currently, no vaccine approved for prophylaxis has been introduced. The treatment strategy for OROV fever relies heavily on the prescription of antipyretic and analgesic medications to relieve the patient's symptoms. Given that the clinical manifestations of OROV infection are very similar to those of diseases caused by arboviruses, such as dengue fever, in their early stages; this has led to an underestimate of the actual number of cases of OROF. OROV, as a neglected virus, is still understudied in terms of its prevalence, transmission, and impact on the epidemiological landscape in Brazil and South America (Andreolla et al., 2024). Currently, OROV infection and its attack on the central nervous system can be recognized early by testing the blood and cerebrospinal fluid of suspected patients. Diagnostic methods include molecular techniques (e.g., RT-PCR or real-time RT-PCR) and traditional virologic assays (e.g., virus isolation, hemagglutination inhibition, and complement-binding assays), but these methods are primarily applicable during the period of viremia (Travassos da Rosa et al., 2017). There are no specific therapeutic drugs or preventive antiviral drugs for OROV infection. Therefore, control or eradication of arthropod vectors and personal protective measures have become the main means of preventing and controlling OROV infection. The risk of OROV infection is particularly significant in midge breeding sites near human settlements. Thus, it is particularly important to strengthen control and personal protection in these areas (Mohapatra et al., 2024). In light of this situation, the relevant authorities, medical experts, research teams, and the international community must come together and implement a proactive, coordinated response.

Our bibliometric analyses play a central role in highlighting the core values of research within a specific area. By comprehensively assessing and integrating research results, we provide insights into research dynamics, prominent contributors, and thematic clusters, thereby enhancing our overall understanding of the knowledge landscape. In the context of OROV research, bibliometric analysis has emerged as a crucial tool for assessing the breadth and impact of scholarly contributions. It not only offers a macroscopic view of the current body of knowledge but also helps identify gaps in knowledge and offers guidance for future research directions. This underscores the significance of bibliometric analysis as a driving force in scientific research and clinical practice, promoting scientific inquiry and enabling well-informed decision-making (Ellegaard and Wallin, 2015).

In an in-depth dissection of the bibliometric analysis of OROV, this study is based on the highly trusted Scopus database, which aims to guarantee the study's rigor. However, this strategy is also accompanied by some inherent limitations. There may be issues of omission of key literature from journals or databases other than SCI, as well as difficulties in fully assessing the quality of newly published literature. Notably, the number of citations is usually accumulated over time, which may result in older literature receiving more citations, while newer studies may be undervalued as a result. Although these limitations may have a subtle impact on the results of the study, they are not sufficient to reverse the major trends and findings of this study on OROV. In addition, this study was limited by the fact that only English language literature in the Scopus database was searched, which may have overlooked equally valuable studies in other languages. In conducting the bibliometric analysis, although we were able to construct quantitative descriptive maps of countries, journals, articles, authors, and keywords about OROV. However, there are still challenges to be faced, such as the difficulty of open access to scientific metrics data, possible incompleteness or duplication of data, and the complexity and diversity of bibliographic data itself. Therefore, researchers need to remain cautious when analyzing data. A deeper understanding of the various dimensions of the data and full consideration of the number of citations with time are required to avoid misjudging the results of recent research.

## Data Availability

The original contributions presented in the study are included in the article/supplementary material, further inquiries can be directed to the corresponding authors.
